# Beyond Classification: An Antineutrophil Cytoplasmic Antibody-Associated Vasculitis Overlap Case

**DOI:** 10.7759/cureus.103402

**Published:** 2026-02-11

**Authors:** Lina Seffar, Abderrahim Elktaibi, Jalila El Bakkouri, Abdelhamid Naitlhou, Abdelkrim Bahlaoui

**Affiliations:** 1 Biology, Cheikh Khalifa International University Hospital, Mohammed VI University of Health Sciences, Casablanca, MAR; 2 Pathology, Cheikh Khalifa International University Hospital, Mohammed VI University of Health Sciences, Casablanca, MAR; 3 Internal Medicine, Cheikh Khalifa International University Hospital, Mohammed VI University of Health Sciences, Casablanca, MAR; 4 Pulmonology, Cheikh Khalifa International University Hospital, Mohammed VI University of Health Sciences, Casablanca, MAR

**Keywords:** anca-associated vasculitis, churg-strauss syndrome, eosinophilia, eosinophilic granulomatosis with polyangiitis, granulomatosis with polyangiitis, overlap syndrome, vasculitis spectrum, wegener’s granulomatosis

## Abstract

Antineutrophil cytoplasmic antibody (ANCA)-associated vasculitides are usually classified as distinct entities, such as granulomatosis with polyangiitis (GPA) and eosinophilic granulomatosis with polyangiitis (EGPA). In everyday practice, however, some patients display overlapping features of both conditions, making classification and treatment decisions more challenging.

We report a case of a 51-year-old man with late-onset asthma who presented with constitutional symptoms, purulent rhinosinusitis, hemoptysis, and arthralgia. Imaging demonstrated cavitary pulmonary nodules and nasal polyposis. Laboratory testing showed marked eosinophilia and positivity for proteinase 3 (PR3) c-ANCA. Nasal biopsy revealed necrotizing granulomatous inflammation rich in eosinophils. The patient received induction therapy with high-dose glucocorticoids and cyclophosphamide, followed by rituximab for maintenance, with clinical improvement and sustained remission. This case highlights the limitations of current classification frameworks and is compatible with a GPA-EGPA overlap phenotype (or spectrum). It underscores the value of an individualized approach guided by the predominant organ-threatening manifestations and the associated biological profile.

## Introduction

Vasculitides are inflammatory disorders of blood vessel walls and are classified according to vessel size and clinicopathologic features, in line with the revised 2012 International Chapel Hill Consensus Conference (CHCC) nomenclature [1]. This framework distinguishes large-, medium-, and small-vessel vasculitides; within small-vessel disease, antineutrophil cytoplasmic antibody (ANCA)-associated vasculitides (AAV) include granulomatosis with polyangiitis (GPA; formerly Wegener’s granulomatosis), eosinophilic granulomatosis with polyangiitis (EGPA; formerly Churg-Strauss syndrome), and microscopic polyangiitis.

The 2022 American College of Rheumatology/European Alliance of Associations for Rheumatology (ACR/EULAR) classification criteria further refine this approach by using data-driven, weighted items applied after confirmation of small- or medium-vessel vasculitis [2,3]. Classification toward GPA is supported by proteinase 3 (PR3)-ANCA positivity, destructive ear-nose-throat (ENT) involvement, and cavitary pulmonary nodules, whereas EGPA is favored by asthma and eosinophilia; myeloperoxidase-antineutrophil cytoplasmic antibodies (MPO-ANCA) contribute variably depending on the scoring profile.

For epidemiologic consistency, the EMA/Watts algorithm assigns each patient hierarchically to a single vasculitis category by integrating ACR and CHCC frameworks, thereby minimizing multiple assignments [4]. Importantly, neither CHCC 2012 nor the 2022 ACR/EULAR criteria define a formal "overlap" category within AAV; in research settings, patients are generally expected to meet one set of classification criteria at a time. Because these criteria were designed for classification rather than diagnosis, they may not capture the full clinical heterogeneity seen in practice.

Consequently, overlapping phenotypes can be encountered. Some patients simultaneously display GPA-like features (PR3-ANCA positivity, destructive sinonasal disease, cavitary pulmonary nodules) and EGPA-like manifestations (late-onset asthma, eosinophilia, nasal polyposis). Although uncommon, these presentations, often described as "polyangiitis overlap syndromes," underscore diagnostic complexity and may require tailored therapeutic strategies.

Here, we report a PR3 c-ANCA-positive patient with ENT and pulmonary involvement occurring in the setting of asthma and eosinophilia who fulfills both the GPA and EGPA 2022 ACR/EULAR classification criteria, illustrating an overlap phenotype and its therapeutic implications.

## Case presentation

A 51-year-old man with allergic rhinitis and late-onset asthma (since his 30s) presented with a two-month history of progressively worsening dyspnea, associated with hemoptysis, epistaxis, and persistent purulent rhinorrhea, along with constitutional symptoms. His asthma was treated with inhaled corticosteroid/long-acting beta-agonist (ICS/LABA) maintenance therapy and short-acting beta-agonist (SABA) as needed, and his rhinitis with an intranasal corticosteroid spray. He had not received systemic corticosteroids or other immunosuppressive therapy prior to admission. He was a former smoker (20 pack-years). Physical examination showed conjunctival injection, a tender submandibular nodule, bilateral wheezes and rhonchi, and polyarthralgia of the elbows and knees, without palpable purpura or peripheral neurologic deficit.

**Figure 1:**
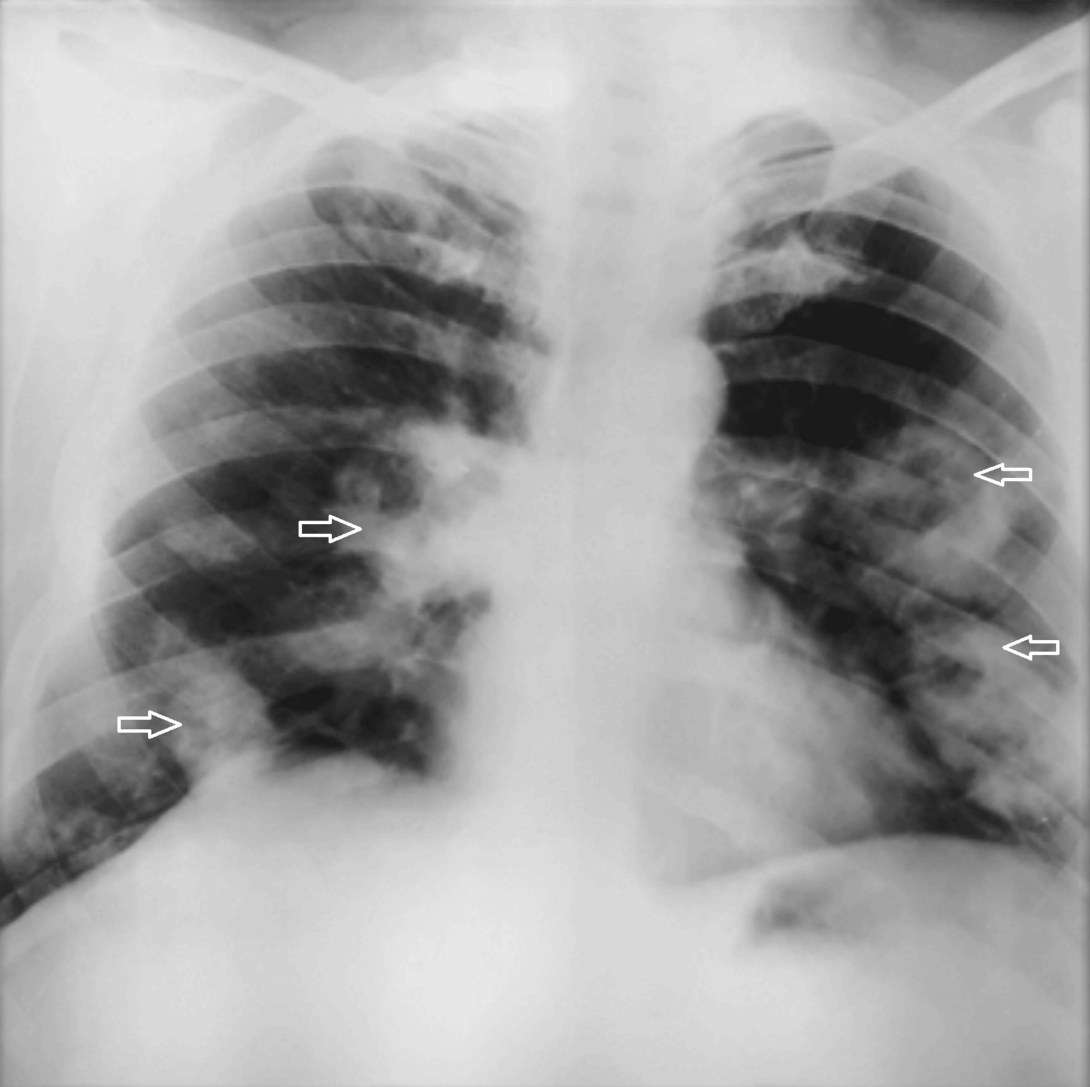
Upright posteroanterior chest radiograph showing multiple bilateral alveolar opacities and areas of consolidation. Arrows indicate opacities with suspected cavitation.

**Figure 2:**
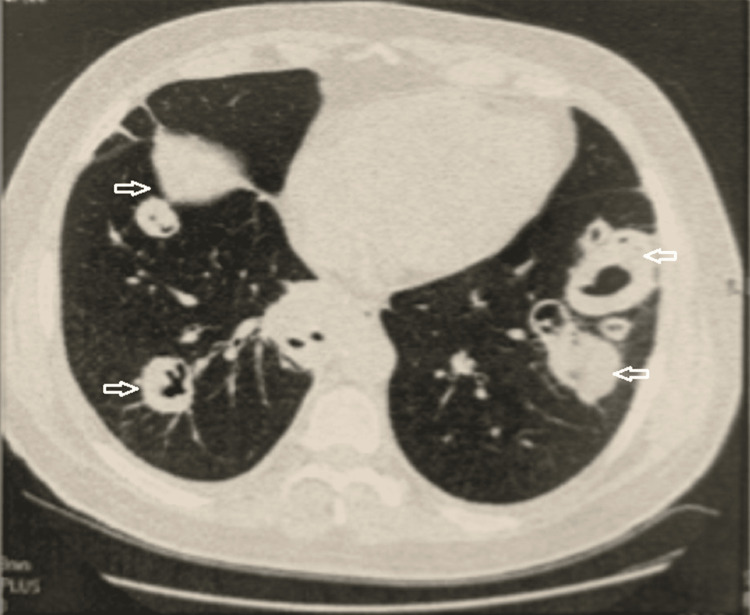
Axial chest CT scan demonstrating multiple bilateral cavitary pulmonary nodules with irregular margins, suggestive of necrosis. Arrows indicate representative cavitary nodules with thick, irregular walls of variable size.

Initial laboratory investigations are summarized in Table 1. Overall, the patient had normocytic, normochromic anemia with leukocytosis and significant eosinophilia, thrombocytosis, and a considerably elevated C-reactive protein level. ANCA testing showed a cytoplasmic pattern with elevated anti-proteinase 3 antibodies and negative anti-myeloperoxidase antibodies. Antinuclear antibodies were positive, and rheumatoid factor was elevated, without clinical features of connective tissue disease or rheumatoid arthritis, and were considered non-specific in this inflammatory context. Total IgE levels were elevated, while the pneumallergen panel and Aspergillus serology were negative. Urinalysis revealed microscopic hematuria with moderate proteinuria, and renal function was preserved. Pulmonary function testing demonstrated a reversible obstructive ventilatory defect consistent with asthma. Chest imaging showed multiple bilateral cavitary pulmonary nodules (Figures 1, 2).

**Table 1:** Laboratory investigations. ANA: antinuclear antibodies; c-ANCA: cytoplasmic ANCA; eGFR: estimated glomerular filtration rate; MPO: myeloperoxidase; MCHC: mean corpuscular hemoglobin concentration; MCV: mean corpuscular volume; PR3: proteinase 3

Parameters	Result	Reference range
Hemoglobin (g/L)	107	130-170
MCV (fL)	83	82-98
MCHC (g/L)	326	320-360
White blood cell count (×10⁹/L)	17.31	4.0-10.0
Eosinophils (×10⁹/L)	1.7 (10%)	<0.46
Platelets (×10⁹/L)	513	150-400
CRP (mg/L)	172	<5
ANA	Positive (1:160)	Negative (<1:80)
c-ANCA	Positive (1:40)	Negative (<1:20)
Anti-PR3 antibodies (U/L)	24	<12
Anti-MPO antibodies (U/L)	<3	<12
Rheumatoid factor (IU/mL)	196	<12.5
Total IgE (IU/mL)	168	<100
Pneumallergen panel	Negative	Negative
Aspergillus serology	Negative	Negative
Hematuria (urine RBC count) (RBC/µL)	20,000	<10
24-hour proteinuria (g/24 h)	0.340	<0.150
eGFR (mL/min/1.73 m²)	89	>60

Sinus computed tomography demonstrated nasal polyposis. Bronchoscopy showed whitish mucosal elevations; transbronchial biopsies revealed intra-alveolar hemorrhage with non-specific chronic fibro-inflammatory changes. Mycological studies and testing for *Mycobacterium tuberculosis *were negative. Allergic bronchopulmonary aspergillosis (ABPA) was considered but deemed unlikely given negative Aspergillus serology, only mildly elevated total IgE, and no bronchiectasis on chest imaging. Parasitic infection was not supported, as parasitological examination of the bronchoalveolar lavage was negative. Primary hypereosinophilic syndrome was also considered; however, biopsy-proven necrotizing granulomatous small-vessel vasculitis together with the overall clinical phenotype supported ANCA-associated vasculitis with eosinophilic features, rather than a primary eosinophilic disorder.

**Figure 3:**
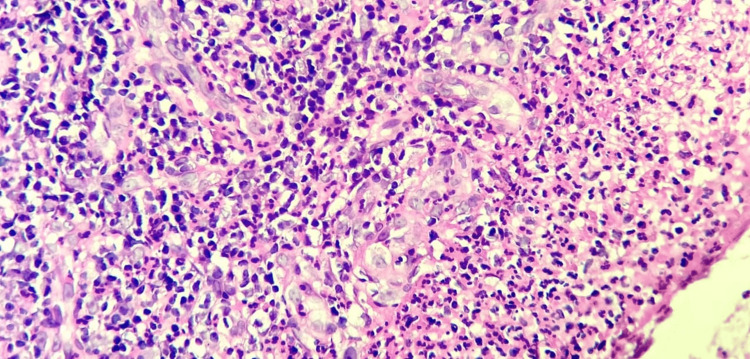
Microphotography showing necrotizing vasculitis and extravascular eosinophil-rich granulomatous inflammation (hematoxylin-eosin staining 200x).

Ear-nose-throat (ENT) involvement was further documented by nasal endoscopy, which showed purulent, edematous, granulomatous rhinosinusitis. Definitive histologic confirmation was obtained from targeted sinonasal biopsies, which demonstrated necrotizing extravascular granulomatous inflammation rich in eosinophils and neutrophils, compatible with small-vessel vasculitis (Figure 3). While the necrotizing pattern could suggest GPA, the prominent eosinophilic infiltrate was atypical for GPA and more suggestive of EGPA. This combination of morphologic features supported the hypothesis of a GPA-EGPA overlap phenotype.

**Figure 4:**
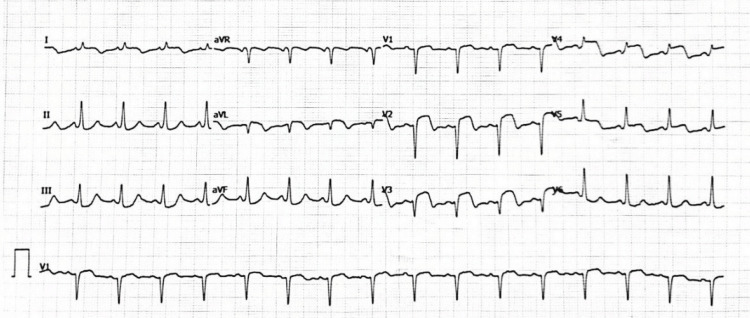
ECG during chest pain showing anterior ST-segment elevation. ST-segment elevation in the anterior precordial leads (V2-V4), with reciprocal inferior changes, consistent with an acute coronary syndrome.

During hospitalization, the patient had an acute coronary syndrome with transient ST-segment elevation and elevated troponin (24.9 ng/mL) (Figure 4). Coronary angiography showed no obstructive coronary lesions, consistent with myocardial infarction with non-obstructive coronary arteries (MINOCA). Alternative causes of MINOCA, including stress (Takotsubo) cardiomyopathy and myocarditis, were considered. Takotsubo was less likely given a reportedly normal transthoracic echocardiogram, whereas myocarditis could not be definitively excluded without cardiac magnetic resonance imaging. In the context of systemic vasculitis, inflammatory coronary involvement and/or coronary vasospasm were also considered.

Disease activity at presentation was high, with a baseline Birmingham Vasculitis Activity Score (BVAS) (v3) score of 31, supporting severe multi-organ disease activity. Accordingly, induction therapy was initiated with intravenous methylprednisolone (1 g/day for three days), followed by oral prednisone (60 mg/day), combined with cyclophosphamide infusions administered once a month.

Over an eight-month follow-up, the patient showed marked clinical improvement, with resolution of ENT and respiratory symptoms and an improved general condition. Follow-up laboratory testing showed a decrease in eosinophilia (from 1.7×10⁹/L to 0.5×10⁹/L) and inflammatory markers (CRP decreased to 10 mg/L). The patient remained in complete remission on maintenance rituximab therapy. Beyond this period, the patient was lost to follow-up.

## Discussion

The 2022 ACR/EULAR classification criteria represent a major step forward compared with the 1990 ACR criteria and subsequent approaches, with overall high performance (sensitivity ~92.5% and specificity ~93.8%) [2,3]. Designed for research classification after confirmation of small- or medium-vessel vasculitis, they rely on a weighted scoring system to better distinguish GPA, MPA, and EGPA. When strictly applied to our patient, however, they yield a dual threshold crossing: a high score supporting GPA (PR3-ANCA positivity, destructive ENT involvement, cavitary pulmonary nodules, and necrotizing granulomatous inflammation) and, simultaneously, a high score supporting EGPA (asthma, nasal polyposis, eosinophilia ≥1.0×10^9^/L, and critically extravascular eosinophil-predominant inflammation on biopsy). This latter finding suggests local eosinophil activation (degranulation and cytotoxic tissue injury), which is consistent with the patient’s asthma and sinonasal disease. This "classification paradox" highlights the limitations of a purely algorithmic interpretation, similar to the hierarchical EMA/Watts algorithm, which improves reproducibility and reduces certain ambiguities (notably GPA-MPA) but does not provide a formal category for GPA-EGPA overlap [4]. Several overlap cases have been reported with features comparable to our patient, most often PR3-positive phenotypes accompanied by eosinophilia [5-9]. In our case, the ACR/EULAR 2022 scores were 9 for EGPA and 9 for GPA (Table 2) [2,3].

**Table 2:** Simplified application of the 2022 ACR/EULAR classification criteria to our patient. EGPA: eosinophilic granulomatosis with polyangiitis; GPA: granulomatosis with polyangiitis; ANCA: antineutrophil cytoplasmic antibody; c-ANCA: cytoplasmic ANCA; ENT: ear-nose-throat; PR3: proteinase 3; ACR/EULAR: American College of Rheumatology/European Alliance of Associations for Rheumatology

Criteria	Scores	Present in the patient
EGPA (threshold ≥6)		
Obstructive airway disease	+3	Yes (asthma)
Nasal polyps	+3	Yes
Eosinophils ≥1×10^9^/L	+5	Yes (1.7×10^9^/L)
Extravascular eosinophilic-predominant inflammation on biopsy	+2	Yes (eosinophil-rich ENT biopsy)
c-ANCA or anti-PR3 positivity	-3	Yes
Hematuria	-1	Yes
Total	9	≥6: EGPA classification met
GPA (threshold ≥5)		
Nasal involvement (bloody discharge/crusting/congestion, etc.)	+3	Yes (purulent rhinorrhea/epistaxis)
c-ANCA or anti-PR3 positivity	+5	Yes (both positive)
Pulmonary nodules/mass/cavitation on imaging	+2	Yes (cavitary nodules)
Granuloma/extravascular granulomatous inflammation on biopsy	+2	Yes (pulmonary and sinonasal biopsies)
Sinus involvement on imaging	+1	Yes (granulomatous sinusitis)
Eosinophils ≥1×10^9^/L	-4	Yes
Total	9	≥5: GPA classification met

Mixed presentations, such as this one, may fit better within a spectrum concept for ANCA-associated vasculitis rather than rigid categories; some authors have even suggested that, in certain patients, pathophysiologic trajectories might shift from one vasculitic phenotype to another over time [10]. On the one hand, the "GPA-like" pole is typically characterized by PR3-ANCA, Th1/Th17-skewed immunity, necrotizing granulomatous inflammation, and destructive ENT and pulmonary disease, often accompanied by pauci-immune glomerulonephritis. On the other hand, the "EGPA-like" pole is anchored in IL-5/eosinophil biology and Th2 pathways, with asthma, nasal polyposis, and eosinophilic tissue infiltrates; peripheral neuropathy (mononeuritis multiplex) is common, and in the ANCA-positive subset, renal involvement may occur, though on average it is less frequent and less severe than in GPA [11]. Overlap phenotypes may reflect concurrent activation of these immune programs, with a PR3-driven neutrophilic/vasculitic component occurring in an eosinophil-prone (often atopic) milieu. This framework is consistent with reports of PR3-positive EGPA or GPA with prominent eosinophilia, suggesting biological "blending" rather than mutually exclusive entities.

This framework is supported by reports of PR3-positive EGPA subgroups whose phenotype resembles PR3-ANCA-associated vasculitides (AAV) biology, with features closer to GPA than to "classic" eosinophilic EGPA (e.g., destructive ENT disease, cavitation, pauci-immune glomerulonephritis). As highlighted by several authors, including Papo et al., these patients may lie along the PR3-AAV spectrum rather than represent a distinct entity [12]. One hypothesis is that a subset of EGPA could develop anti-PR3 antibodies while retaining an eosinophilic background, potentially influenced by autoimmune genetic susceptibility. Variants within HLA-DPB1 have been associated with PR3-AAV and with T-cell responses directed against PR3. In simple terms, some patients may be genetically predisposed to a PR3 (GPA-like) immune signature; this predisposition could coexist with an atopic/eosinophilic background and contribute to the emergence of mixed phenotypes [13]. However, these associations reflect population-level risk and do not establish causality for overlap phenotypes; effect sizes and lead variants can vary across ancestries, and extrapolation to GPA-EGPA overlap remains speculative. Therefore, we mention HLA-DPB1 as a biologically plausible hypothesis rather than a definitive explanation.

Conversely, eosinophilia is not exclusive to EGPA and can be observed in GPA [12,14,15]. A French cohort reported moderate eosinophilia in approximately one quarter of GPA patients, with severe hypereosinophilia in a smaller subset; this was associated with more intense systemic inflammation, suggesting eosinophil-driven tissue injury in selected phenotypes [15]. At the same time, eosinophilia remains a cardinal hallmark of EGPA [16]. In this context, some authors have proposed that overlap syndromes may reflect PR3-positive vasculitis expressed on an atopic background, reflecting an interaction between neutrophil-driven autoimmunity and eosinophilic predisposition [17]. Overall, these data emphasize the heterogeneity of GPA-EGPA overlap presentations and support the concept of an overlap phenotype or a distinct subtype with both pathogenic and therapeutic implications.

From a practical standpoint, the goal is not necessarily to force a single label (GPA vs. EGPA), but to identify the dominant organ-threatening phenotype to guide induction therapy, and then adjust treatment to address the secondary component. In our patient, cavitary pulmonary nodules with hemoptysis, destructive ENT involvement, and a myocardial infarction with non-obstructive coronary arteries (MINOCA)/ST-elevation presentation in the setting of PR3-ANCA positivity supported a severe vasculitic (PR3-AAV-like) phenotype with high baseline activity (BVAS 31). Accordingly, induction with high-dose glucocorticoids plus cyclophosphamide was selected; however, rituximab would represent a reasonable alternative induction strategy in PR3-ANCA-positive AAV, depending on local practice and patient-specific factors. After remission, rituximab maintenance was favored given the relapse propensity typically associated with PR3-ANCA disease, with follow-up focusing on both vasculitic activity (ENT/pulmonary/renal) and the eosinophilic component (asthma control, eosinophil counts). Anti-IL-5/IL-5R therapy (e.g., mepolizumab or benralizumab) is an important option in EGPA when eosinophil-driven manifestations predominate or remain refractory, particularly asthma/nasal polyposis, or when suspected eosinophilic tissue injury is present. However, it was not prioritized here because the presentation was primarily vasculitic and responded promptly to standard induction therapy. Overall, these cases highlight the need for dynamic, phenotype-based management and periodic reassessment as the clinical picture may evolve over time. Because overlap phenotypes may prompt escalation of immunosuppression, treatment should remain severity- and phenotype-driven, balancing organ-threatening disease control against treatment-related risks (serious infection, glucocorticoid toxicity, and cyclophosphamide-related adverse effects), with tapering and de-escalation once remission is achieved.

This case also highlights priorities for future work. Prospective cohorts with standardized definitions of "overlap" phenotypes are needed to determine whether these presentations represent a distinct subgroup or a continuum within ANCA-associated vasculitis. Biomarker-driven models integrating ANCA specificity (PR3 vs. MPO) with eosinophil/Th2 activity markers (eosinophil counts, IL-5-related signatures, asthma/nasal polyposis, and tissue eosinophilia) may better capture mixed biology than mutually exclusive labels. Such approaches could ultimately inform refinement of classification frameworks and help tailor treatment strategies for patients with blended vasculitic and eosinophilic features.

## Conclusions

This case illustrates the complexity of mixed presentations combining features of granulomatosis with polyangiitis and eosinophilic granulomatosis with polyangiitis, which can challenge current classification frameworks. It highlights the importance of a comprehensive clinical assessment and an individualized therapeutic strategy guided by the dominant phenotype and the patient’s biological course over time. Recognizing these overlapping presentations suggests that a subset of ANCA-associated vasculitides may be better understood along a clinical spectrum rather than as strictly separate entities.

## References

[1] Jennette JC, Falk RJ, Bacon PA (2013). 2012 revised International Chapel Hill Consensus Conference Nomenclature of Vasculitides. Arthritis Rheum.

[2] Robson JC, Grayson PC, Ponte C (2022). 2022 American College of Rheumatology/European Alliance of Associations for Rheumatology classification criteria for granulomatosis with polyangiitis. Ann Rheum Dis.

[3] Grayson PC, Ponte C, Suppiah R (2022). 2022 American College of Rheumatology/European Alliance of Associations for Rheumatology classification criteria for eosinophilic granulomatosis with polyangiitis. Ann Rheum Dis.

[4] Watts R, Lane S, Hanslik T (2007). Development and validation of a consensus methodology for the classification of the ANCA-associated vasculitides and polyarteritis nodosa for epidemiological studies. Ann Rheum Dis.

[5] Bruno L, Mandarano M, Bellezza G, Sidoni A, Gerli R, Bartoloni E, Perricone C (2023). Polyangiitis overlap syndrome: a rare clinical entity. Rheumatol Int.

[6] Leavitt RY, Fauci AS (1986). Polyangiitis overlap syndrome. Classification and prospective clinical experience. Am J Med.

[7] Quan MV, Frankel SK, Maleki-Fischbach M, Tan LD (2018). A rare case report of polyangiitis overlap syndrome: granulomatosis with polyangiitis and eosinophilic granulomatosis with polyangiitis. BMC Pulm Med.

[8] Uematsu H, Takata S, Sueishi K, Inoue H (2014). Polyangiitis overlap syndrome of granulomatosis with polyangiitis (Wegener's granulomatosis) and eosinophilic granulomatosis with polyangiitis (Churg-Strauss syndrome). BMJ Case Rep.

[9] Subramanian A, Hanchosky A, Vuyyuru S, Coffey K, Massri T, Stewart C (2023). A rare presentation of polyangiitis overlapping syndrome. Cureus.

[10] Zia Z, Renz JJ, Sammar A, Gordon JR (2023). EGPA imitating GPA: a misdiagnosis or evolving pathophysiology?. Chest.

[11] Zeleke ST, Zaidi SR, Gebremedhen AI, Becker M (2025). Overlap or outlier? Granulomatosis with polyangiitis with eosinophilia: a case report and diagnostic insight. J Investig Med High Impact Case Rep.

[12] Papo M, Sinico RA, Teixeira V (2021). Significance of PR3-ANCA positivity in eosinophilic granulomatosis with polyangiitis (Churg-Strauss). Rheumatology (Oxford).

[13] Merkel PA, Xie G, Monach PA (2017). Identification of functional and expression polymorphisms associated with risk for antineutrophil cytoplasmic autoantibody-associated vasculitis. Arthritis Rheumatol.

[14] Shoda H, Kanda H, Tanaka R, Komagata Y, Misaki Y, Yamamoto K (2005). Wegener's granulomatosis with eosinophilia. Intern Med.

[15] Iudici M, Puéchal X, Pagnoux C (2022). Significance of eosinophilia in granulomatosis with polyangiitis: data from the French Vasculitis Study Group Registry. Rheumatology (Oxford).

[16] Comarmond C, Pagnoux C, Khellaf M (2013). Eosinophilic granulomatosis with polyangiitis (Churg-Strauss): clinical characteristics and long-term followup of the 383 patients enrolled in the French Vasculitis Study Group cohort. Arthritis Rheum.

[17] Abida H, Somaï M, Rachdi I (2022). Diagnostiquer une granulomatose avec polyangéite: à travers les différents scores de classification. [Article in French]. Rev Méd Interne.

